# Application of 2,4-Epibrassinolide Improves Drought Tolerance in Tobacco through Physiological and Biochemical Mechanisms

**DOI:** 10.3390/biology11081192

**Published:** 2022-08-08

**Authors:** Rayyan Khan, Xinghua Ma, Quaid Hussain, Muhammad Asim, Anas Iqbal, Xiaochun Ren, Shahen Shah, Keling Chen, Yi Shi

**Affiliations:** 1 Key Laboratory of Tobacco Biology and Processing, Ministry of Agriculture and Rural Affairs, Tobacco Research Institute, Chinese Academy of Agricultural Sciences, Qingdao 266101, China; 2 State Key Laboratory of Subtropical Silviculture, Zhejiang A&F University, 666 Wusu Street, Hangzhou 311300, China; 3 College of Life Science and Technology, Guangxi University, Nanning 530004, China; 4 Department of Agronomy, The University of Agriculture Peshawar, Peshawar 25130, Pakistan

**Keywords:** 2,4-epibrassinolide, brassinosteroids, drought tolerance, drought stress, cell expansion and leaf thickness, brassinosteroids and auxin signaling

## Abstract

**Simple Summary:**

Drought stress is one of the most serious abiotic stresses and negatively affects crop growth and development. Given global climate change, it is important to identify effective methods of alleviating drought stress effects. Brassinosteroids (2,4-epibrassinolide-EBR) play an important role in mitigating the negative effects of drought stress on plants. Therefore, this study evaluated the role of EBR in improving drought tolerance. The results demonstrated that EBR application improved drought tolerance by strengthening the enzymatic antioxidant defense system and osmoregulation to scavenge reactive oxygen species. EBR application improved BR and IAA phytohormone content and improved drought tolerance by upregulating genes related to their respective signaling pathways. Therefore, EBR application is an effective strategy for improving drought tolerance in crop plants.

**Abstract:**

Drought stress is a major abiotic stress that hinders plant growth and development. Brassinosteroids (BR), including 2,4-epibrassinolide (EBR), play important roles in plant growth, development, and responses to abiotic stresses, including drought stress. This work investigates exogenous EBR application roles in improving drought tolerance in tobacco. Tobacco plants were divided into three groups: WW (well-watered), DS (drought stress), and DSB (drought stress + 0.05 mM EBR). The results revealed that DS decreased the leaf thickness (LT), whereas EBR application upregulated genes related to cell expansion, which were induced by the BR (*DWF4*, *HERK2*, and *BZR1*) and IAA (*ARF9*, *ARF6*, *PIN1*, *SAUR19*, and *ABP1*) signaling pathway. This promoted LT by 28%, increasing plant adaptation. Furthermore, EBR application improved SOD (22%), POD (11%), and CAT (5%) enzyme activities and their related genes expression (*FeSOD*, *POD*, and *CAT*) along with a higher accumulation of osmoregulatory substances such as proline (29%) and soluble sugars (14%) under DS and conferred drought tolerance. Finally, EBR application augmented the auxin (IAA) (21%) and brassinolide (131%) contents and upregulated genes related to drought tolerance induced by the BR (*BRL3* and *BZR2*) and IAA (*YUCCA6*, *SAUR32*, and *IAA26*) signaling pathways. These results suggest that it could play an important role in improving mechanisms of drought tolerance in tobacco.

## 1. Introduction

Global climate change poses a major threat to plant survival and has disturbed the growth patterns of plants [1]. Climate change has increased the occurrence and frequency of abiotic stresses, including drought stress (DS) [2]. Due to the sessile nature of plants, they face different abiotic stresses that are hostile to plant growth and development [3]. Drought stress is one of the most severe and inevitable factors hampering plant growth and development [4], and affects 45% of global agricultural land [5]. DS hinders plant growth and development by decreasing plant biomass and chlorophyll content and increasing levels of reactive oxygen species (ROS) and malondialdehyde (MDA) content [6]. Stressful conditions unbalance the equilibrium between ROS production and the antioxidant defense system, leading to overproduction of ROS and causing oxidative damage and, ultimately, cell death [7]. The equilibrium between ROS generation and detoxification is maintained by the plant antioxidant defense system (enzymatic and non-enzymatic) under stressful conditions [8]. The enzymatic antioxidant defense system comprises superoxide dismutase (SOD), peroxidase (POD), and catalase (CAT), whereas the non-enzymatic antioxidant defense system contains ascorbic acid (AsA) and proline [9].

Leaf thickness is the distance between the adaxial and abaxial surfaces and is an important morphological trait contributing to plant and leaf functioning [10]. The leaf thickness is correlated to various environmental variables. Several published studies shed light on the response of leaf thickness to various abiotic stresses; for example, salt stress reduced the thickness of the upper epidermis, mesophyll tissues (palisade and spongy cells), and ultimately leaf thickness [11]. The leaf anatomy of tobacco seedlings is changed, and leaf thickness is altered by drought stress [12]. Drought stress decreased the leaf thickness in tomato [13] and peanut [14]. In another study, *Platanus orientalis* plants in two different habitats were evaluated under drought stress, drier (IT—Italy) and more humid (BG—Bulgaria), and drought stress increased leaf thickness shrinkage in BG plants, whereas after re-watering, IT plants retained more water by maintaining thicker leaves than BG plants [15]. Therefore, leaf thickness can be used as a drought tolerance indicator [16].

Brassinosteroids (BR) are a major class of growth-promoting hormones that regulate plant growth and development in many aspects of plant life, such as cell elongation and responses to various environmental stresses, including drought stress [17,18]. Several studies have shown that BR application confers drought tolerance via higher antioxidant enzyme activities such as SOD, POD, and CAT, along with a higher accumulation of proline, and consequently lowered the ROS production and MDA content [19,20,21]. 2,4-epibrassinolide (EBR) application proved as an effective stress ameliorating approach. Several studies highlighted the potential role of EBR application in conferring various abiotic stress tolerance including drought stress [22], salt stress [23], heat stress [24], and heavy metals stress [25] by keeping a balance between ROS and antioxidants and osmolytes accumulation. In addition to its role in responding to stressful conditions, BR is also involved in several growth processes, such as cell division, cell elongation, and vascular differentiation [26]. In a BR-deficient mutant of *Arabidopsis thaliana* (*dwf4*), the BR application from 1 nM to 100 nM promoted cell elongation in the meristem [27]. By laying the foundation of BR involvement in various aspects of plant growth, development, and response to abiotic stresses, auxin (IAA) also play a crucial role in plant growth, development, and stress tolerance [28]. In white clover, the mechanisms related to drought tolerance by IAA are higher phytohormone contents, especially ABA and IAA, and ABA responsive and drought-tolerance-related gene expression [29]. The transgenic studies or loss of functional analysis of genes related to IAA exhibited drought tolerance by modulating the IAA content, metabolite accumulation (organic acids, sugars, sugar alcohols), stress-responsive gene expression, and oxidative damage [30,31,32]. Thus, this study was planned to understand the interaction of BR with IAA in regulating leaf thickness under drought stress.

Tobacco (*Nicotiana tabacum* L.) is a model plant for studying plant biology and genetics and is also an important economic crop [33]. It is vulnerable to drought stress and is affected at different growth stages, such as the seedling, rosette, vigorous, and flowering stages [12,34,35]. The intensity and duration of DS are intensifying due to drastic changes in climatic changes. It is important to improve plant performance under drought stress, given the future of global climate change. Therefore, this study was designed to examine the regulatory role of EBR on leaf anatomy, enzymatic antioxidant defense system, phytohormone content, and gene expression for enhancing tobacco drought tolerance.

## 2. Materials and Methods

### 2.1. Plant Materials, Growth Conditions, and 2,4-Epibrassinolide Treatment

This research was designed to better understand the regulatory roles of EBR in conferring drought tolerance using tobacco (*Nicotiana tabacum* L.) K326 cultivar. This cultivar was introduced in China by the Yunnan Branch of China Tobacco Company (Kunming, China) and selected by Northup King Seed Company (Minneapolis, MN, USA). This variety was given a national serial number “00002266” after passing the DUS (Distinctness, Uniformity, and Stability) test, approval, and certification from the National Tobacco Variety Certification Committee. The K326 seeds were provided by the National Infrastructure for Crop Germplasm Resource Tobacco (Qingdao, China). The experiment was carried out in a growth chamber of the Tobacco Research Institute of the Chinese Academy of Agricultural Sciences. The seeds were sown in small trays, and after germination, the seedlings were transferred to pots containing a mixture of peat and vermiculite (vol/vol, 1:1). The growth chamber conditions for the experiments were: temperature (26 °C), light intensity (300 μmol m^−2^ s^−1^), and photoperiod (15h/9h, day/night). The experiment was performed on the seedlings with uniform growth. The seedlings were then divided into three groups, namely, well-watered (WW), drought stress (DS), and drought stress plus foliar 2,4-Epibrassinolide (EBR) application (DSB). The EBR (purity-98%) was purchased from Solarbio Science and Technology Company Limited (Beijing, China). Based on our previous results [36], we chose 0.05 mM concentration for foliar/exogenous EBR application for this experiment under DS. The DS plants were sprayed with ddH_2_O. In total, 10 mL of EBR solution was sprayed on one plant on the adaxial side of the leaves. The 0.05 mM EBR was sprayed on plants every three days. For WW plants, the moisture content was retained at a field capacity (FC) of 80%, whereas 60% FC for DS was based on daily measurement of pot weight [37]. Drought stress was applied to plants for 24 days. The samples were taken from WW, DS, and DSB at the end of the experiment. The 8th leaf from base to tip was sampled and the biochemical analysis was performed using three biological repeats. Similarly, the gene expression analysis was performed on leaf samples using three biological repeats and three technical repeats. The leaf samples taken from the plants were immediately immersed in liquid nitrogen and stored at −80 °C for further analysis.

### 2.2. Anatomical Analysis

The leaf anatomical analysis was performed by taking leaf samples each from WW, DS, and DSB (*n* = 9). The samples were taken from the mid-portion between the midrib and margin of the leaves and stored in a 50% FAA (formalin: acetic acid: alcohol) solution. Briefly, by following the method of Moreno-Sanz et al. [38] and Faraone et al. [39] with few modifications, the paraffin sections of samples were rinsed two times in xylene (100%) for 20 min. The sections were then rehydrated by keeping them in absolute ethanol and 75% ethanol each for 5 min. After rehydration, dyeing was carried out under light conditions. Safranin O (Servicebio, Wuhan, China) solution was used for dyeing the samples. After dyeing with Safranin O (1%) for 2 hours, the sections were washed with tap water to wash away the excessive dye. Then, rapid dehydration of the sections was performed by rinsing them in graded ethanol series (50%-3–8 s, 70%-3–8 s, and 80%-3–8 s). The sections were again dyed with Fast Green FCF (0.5%) (Servicebio, Wuhan, China) for 6–20 s. Before the final rinsing in the xylene for 5 min, all the sections were again passed from the absolute ethanol three times for rapid dehydration. Furthermore, the leaf sections were mounted on slides using neutral balsam. Finally, the photography of the samples for leaf structure was performed on the slides using the microscope (Leica DM 2000, Wetzlar, Germany) coupled with a Leica DMC 2900 camera (Leica, Wetzlar, Germany). This Leica DM 2000 microscope was designed with halogen lamp as an illumination source (light wavelength—340 to 800 nm). The leaf thickness, thickness of upper epidermis, lower epidermis, spongy cells, and palisade cell length were measured from the acquired images using ImageJ software (http://rsbweb.nih.gov/ij/, accessed on 05 January 2022).

### 2.3. Measurement of Biochemical Parameters

#### 2.3.1. Determination of Reactive Oxygen Species (ROS) and Malondialdehyde (MDA)

The ROS was determined by weighing 0.1 g sample and ground the sample in 1 mL Tris-HCl (50 mM, pH 7.4) buffer containing sucrose and EDTA and 10 μL PMSF solution. After grinding and homogenization, the homogenate was centrifuged at 600 rpm for 5 min at 4 °C. The supernatant was collected in another tube and again centrifuge at 11,000 rpm for 10 min at 4 °C. The supernatant was discarded and 200 μL Tris-HCl buffer containing KCl and MgCl_2_·6H_2_O was added to the tube having the pellet. Then, in 20 μL sample suspension, 50 μL each PMSF solution, malic acid, pyruvate, succinic acid, and 30 μL DCFH-DA solutions were added and incubated in dark for 15 min at 37 °C. After incubation, the absorbance value at respective wavelengths of 499 nm and 521 nm of excitation and emission was recorded within 10 min at a constant temperature of 37 °C.

The MDA content was determined by weighing a 0.1 g sample and ground in 1 mL phosphate-buffered solution (PBS) (100 mM, pH 7.5). After homogenization, the homogenate was centrifuged at 8000 rpm at 4 °C for 10 min. A 0.3 mL TCA and TBA were added to 0.1 mL sample homogenate and mixed well. The sample tubes were incubated in a water bath at 95 °C for 30 min and then cooled to room temperature. The samples were then centrifuged at 25 °C for 10 min using 10,000 rpm. Finally, 200 μL of the sample homogenate was taken and the absorbance was recorded at 532 nm and 600 nm [40].

#### 2.3.2. Determination of Antioxidant Enzyme Activities

The antioxidant enzyme activities were determined by grinding 0.1 g sample in a 1 mL PBS solution. The homogenate was centrifuged for 10 min at 4 °C using 8000 rpm. The supernatant was collected to determine the activities of SOD, POD, and CAT. Their activities were presented as U mg^−1^ protein.

The SOD activity was determined by reading the absorbance at 450 nm. One unit of SOD is the amount of enzyme needed to dismutase 50% of available superoxide radicals [40]. The POD enzyme activity was detected at 470 nm and one unit is the amount of enzyme that changes the absorbance by 0.01 per min of guaiacol oxidation [41]. CAT enzyme activity was determined by reading the absorbance at 240 nm. One unit of CAT is defined as the decomposition of H_2_O_2_ in one min [42].

#### 2.3.3. Determination of Proline and Soluble Sugar Contents

The proline content was determined in the three treatments (WW, DS, and DSB) following the method of Sun et al. [40] by weighing a 0.1 g sample and homogenizing it in a 1 mL sulfosalicylic acid solution. After homogenization, the homogenate was kept in a water bath at 95 °C for 10 min and well shaken during this time. After incubation, the homogenates were then centrifuged at 25 °C for 10 min using 10,000 rpm. The supernatant was collected and kept on ice for further use. Next, the 0.25 mL sample, 0.25 mL glacial acetic acid, and 0.25 mL ninhydrin, glacial acetic acid, and concentrated phosphoric acid solution were placed into a tube and kept in a water bath for 30 min at 95 °C and shook well every 10 min. Then, this mixture was cooled to room temperature. Overall, 0.5 mL toluene was added and shook for 30 s, then left to stand for a while. Then, we took 0.2 mL solution from the upper portion, and the absorbance was recorded at 520 nm.

The soluble sugar content was determined by following the method of Wang et al. [43]. A 0.1 g sample was ground in 1 mL distilled water. The homogenate was centrifuged at 25 °C for 10 min at 8000 rpm. Then, 0.2 mL sample supernatant and distilled water (blank tube) was added to 1 mL anthrone reagent and shook well. The sample and blank tubes were kept in a water bath for 10 min at 95 °C and cooled to room temperature. Finally, the absorbance was recorded at 620 nm.

### 2.4. Phytohormone (IAA and BR) Contents Determination

The IAA content was measured by weighing 0.1 g of frozen samples and grounded in 1 mL of pre-cooled methanol aqueous solution and incubated overnight at 4 °C. On the next day, the samples were centrifuged at the same temperature for 10 min at 8000 rpm and the supernatant was collected. After the supernatant collection, the leftover residues were again extracted by adding 0.5 mL methanol aqueous solution and incubated for 2 h and then centrifuged. The supernatant was again collected and the two supernatants were combined. The supernatant was dried using N_2_ at 40 °C. Next, 0.5 mL petroleum ether was added and the sample fractions (for IAA) were extracted three times. The upper organic phase (ether) was discarded and citric acid aqueous solution (pH 4.5) was added. The sample fraction was extracted three times with ethyl acetate in order to combine the organic phases. The sample fractions were dried under N_2_ and 0.5 mL methanol was added for further analysis. After methanol addition, the sample fractions were vortex-shaken, syringe-filtered, and tested for IAA content. The IAA content was determined using the Rigol L3000 HPLC system (Beijing, China) equipped with Alphasil CV-C_18_ reversed-phase column (4.6 mm × 250 mm, 5 μm). The analysis was performed with an injection volume of 10 μL with a 0.8 mL min^−1^ flow rate at 35 °C column temperature having 40 min aliasing time. The mobile phase for IAA determination comprised ultrapure water (600 mL), acetic acid (6 mL), and methanol (400 mL). The IAA content was measured using the excitation wavelength of 275 nm and emission wavelength of 345 nm [44].

The brassinolide content, a class of brassinosteroids, was determined in the frozen samples (−80 °C) as per guidelines of the Plant Brassinosteroid ELISA Kit (Cat No.2PI kmLJ91188p) which was purchased from Camilo Biological Company Limited (Nanjing, China). Briefly, the tissue homogenate was prepared by grounding 0.2 g sample in 1.5 mL 0.01 M BPS on an ice bath. After being blended and ground, the homogenate was centrifuged for 10 min at 10,000 rpm. The supernatant was collected for further analysis. The blank strips (having wells) took out from the kit (stored at 2–8 °C) and set aside to be balanced to room temperature. A 100 μL sample or different concentrations of BR standard samples were added to corresponding wells and kept in an incubator for 90 min at 37 °C. After incubation, the strips were washed with washing buffer (PBS) two times and 100 μL biotinylated plant BR antibody was added to each well. The reaction wells were sealed with adhesive tapes and the wells were kept in an incubator for 60 min at 37 °C. The reaction wells were washed 3 times with washing buffer after incubation. Following this, 100 μL enzyme conjugate was added to each well and sealed with adhesive tapes. After sealing the wells, these were kept for incubation for 30 min at 37 °C. The reaction wells were then washed 5 times with washing buffer, and 100 µL color reagent liquid was added to each reaction well and sealed for dark incubation at 37 °C for 30 min. Then, 100 μL Stop solution (1M sulfuric acid) was added to each reaction well and mixed well when the color of the high concentration standard became darker and a color gradient appeared. Finally, the OD was read at 450 nm within 10 min after adding the stop solution [45].

### 2.5. RNA Extraction, cDNA Synthesis, and RT-qPCR

In this study, FastPure Plant Total RNA Isolation Kit was used for the extraction of total RNA as per manufacturer guidelines and then cDNA was synthesized following the instructions of HiScript^®^ III SuperMix for qPCR (+gDNA wiper) kit. Both RNA extraction and cDNA synthesis kits were purchased from the Vazyme Biotech Co., Ltd., Nanjing, China. SYBR Premix Ex Taq II (Takara, Japan) master mix was used to perform the RT-qPCR in the Applied Biosystems Quantstudio3 Real-Time PCR machine of Thermo Fisher Scientific (Waltham, MA, USA). The RT-qPCR was performed on three biological repeats while using *Actin* as an internal control, and the data were analyzed using the 2^−∆∆CT^ method [46]. Please see Supplementary Table S1 for the primers used in this study.

### 2.6. Statistical Analysis

The data were collected, arranged, and analyzed by Statistix 8.1 software (Analytical Software, Tallahassee, FL, USA) using one way ANOVA model. After performing ANOVA, LSD test was used to find the significant (*p* < 0.05) differences between the means of the different treatments such as WW, DS, and DSB. Subsequently, the means data of the treatments ± standard error were transformed into graphs using OriginPro 9.1 software package (OriginLab Corporation, Northampton, MA, USA).

## 3. Results

### 3.1. Changes in Leaf Anatomy

**Figure 1:**
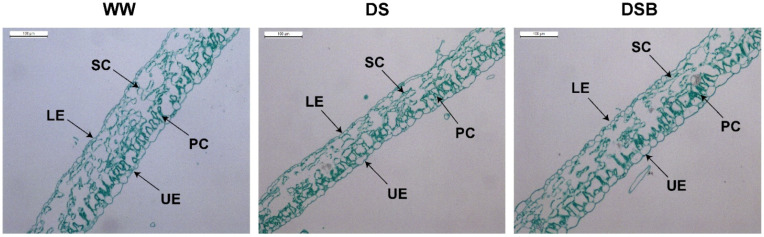
Representative leaf structure images under 2,4-epibrassinolide (EBR) application in response to drought stress. WW (well-watered), DS (drought stress), and DSB (drought stress + 0.05 mM EBR application) are the three treatments. LE—lower epidermis, UE—upper epidermis, PC—palisade cells, SC—spongy cells.

**Figure 2:**
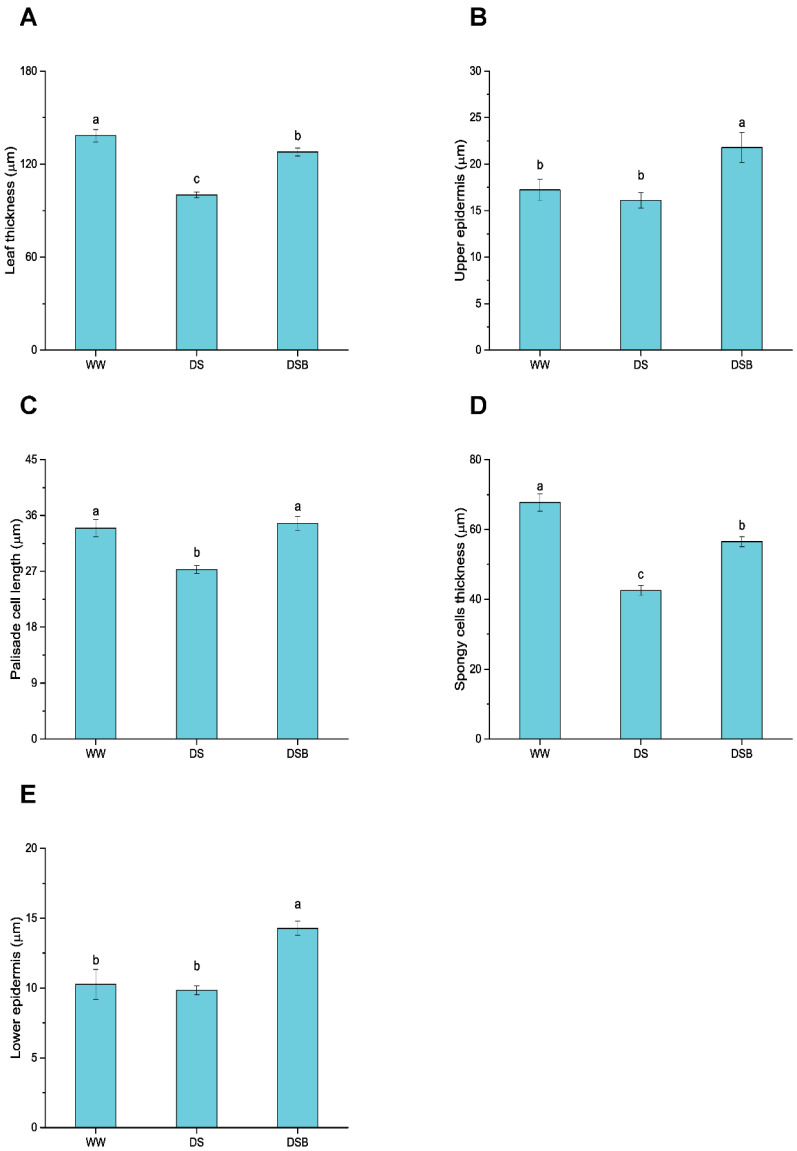
Leaf structure is regulated by 2,4-epibrassinolide (EBR) application under drought stress. This figure displays the total leaf thickness (**A**), upper epidermis thickness (**B**), palisade cells length (**C**), spongy tissue thickness (**D**), and lower epidermis thickness (**E**). The data are presented as means ± SE in the three treatments including WW (well-watered), DS (drought stress), and DSB (drought stress + 0.05 mM EBR application). Error bars represent SE of the mean. Different letters on bars show significant differences at *p* < 0.05 using the LSD test.

Images of the representative leaf structure from the three treatments (WW, DS, and DSB) are presented in Figure 1 and show clear differences between the treatments. We noted a clear decrease in the leaf thickness in DS compared with WW and an increase in DSB compared with DS (Figure 1). The leaf thickness, upper epidermis thickness (UE), palisade cell length (PCL), spongy tissue thickness (STT), and lower epidermis thickness (LE) were significantly (*p* < 0.05) affected by EBR application under drought stress. The leaf thickness was decreased both in DS (100.05 μm) and DSB (127.82 μm) treatments by 28% and 8%, respectively, compared with WW (138.36 μm). However, a 28% increase in leaf thickness was observed in DSB compared with DS (Figure 2A). The UE was thickened by 26% and 35% in DSB (21.77 μm) compared with WW (17.22 μm) and DS (16.10 μm), respectively, whereas a nonsignificant decrease of 7% was noted in DS compared with WW (Figure 2B). Among the mesophyll cell layers, the PCL was 20% lower in DS (27.28 μm) compared with WW (33.95 μm) and was 27% thicker in DSB (34.74 μm) compared with DS (Figure 2C). The STT showed respective reductions of 37% and 17% in DS (42.49 μm) and DSB (56.51 μm) compared with WW (67.76 μm) and was 33% thicker in DSB compared with DS (Figure 2D). Finally, the LE showed a 39% and 45% increase in its thickness in DSB (14.28 μm) compared with WW (10.26 μm) and DS (9.84 μm), respectively (Figure 2E).

### 3.2. Cell-Expansion-Related Gene Expression via BR and IAA Signaling Pathways

**Figure 3:**
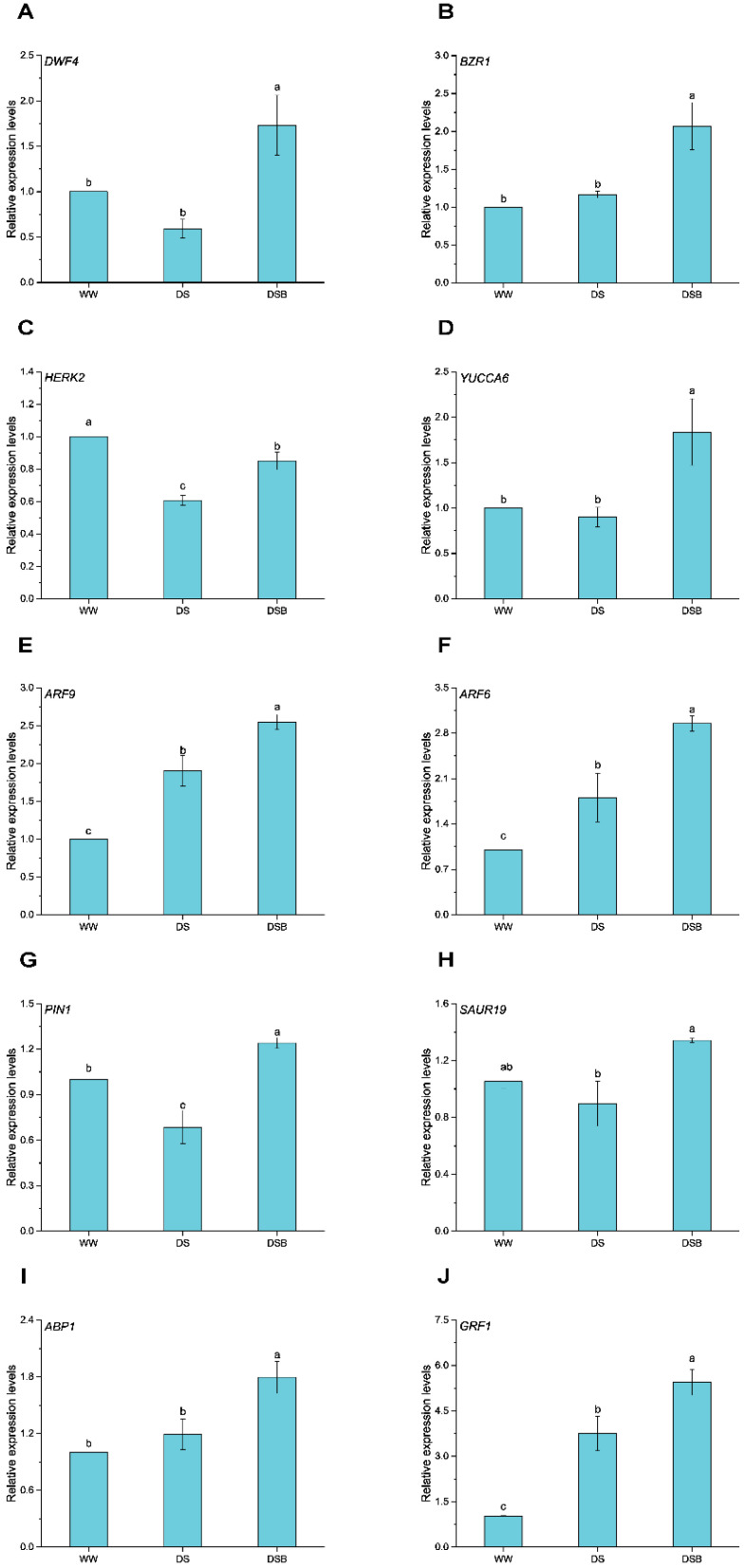
Cell-expansion-related gene expression is mediated by the BR and IAA signaling pathways. This figure comprises gene expressions such as *DWF4* (**A**), *BZR1* (**B**), *HERK2* (**C**), *YUCCA6* (**D**), *ARF9* (**E**), *ARF6* (**F**), *PIN1* (**G**), *SAUR19* (**H**), *ABP1* (**I**), and *GRF1* (**J**). The data are presented as means ± SE in the three treatments including WW (well-watered), DS (drought stress), and DSB (drought stress + 0.05 mM 2,4-epibrassinolide application). Error bars represent SE of the mean; different letters on bars show significant differences at *p* < 0.05 using the LSD test.

The expression of genes related to BR biosynthesis, signaling, and BR regulated-cell expansion (*DWF4*, *BZR1*, and *HERK2*), and IAA biosynthesis, signaling, and IAA regulated-cell expansion (*YUCCA6, ARF9, ARF6*, *PIN1, SAUR19*, *ABP1*, and *GRF1*) were significantly (*p* < 0.05) affected by EBR application under drought stress (Figure 3). *DWF4* was significantly upregulated only in DSB by 1.7 and 2.9 times compared with WW and DS, respectively (Figure 3A). *BZR1* was also significantly upregulated only in DSB and showed expression levels that were 2.1 and 1.8 times higher than WW and DS, respectively (Figure 3B). *HERK2* was downregulated in DS and DSB compared with WW; however, EBR increased its expression by 1.4 times compared with DS (Figure 3C). *YUCCA6* was upregulated in DSB by 1.8 and 2.0 times compared with WW and DS, respectively (Figure 3D). The *ARF9* showed a 1.9-times higher expression in DS compared with WW and a 2.5- and 1.3-fold higher expression in DSB compared with WW and DS, respectively (Figure 3E). *ARF6* was upregulated both under DS and DSB compared with WW. Its transcript levels were 1.8 and 2.9 times higher in DS and DSB compared with WW, respectively, and 1.6 times higher in DSB than DS (Figure 3F). *PIN1* was downregulated by 0.7-fold in DS compared with WW, and was upregulated in DSB by 1.2- and 1.8-fold higher expression compared with WW and DS, respectively (Figure 3G). Moreover, *SAUR19* showed an expression that was 1.5-fold higher in DSB than DS and was found not significant between DSB and WW (Figure 3H). Additionally, *ABP1* was only significantly upregulated in DSB and its expression was increased by 1.8- and 1.5-fold compared to WW and DS, respectively (Figure 3I). *GRF1* was upregulated in both DS (3.7 times) and DSB (5.3 times) compared to WW. Of note, its expression was 1.5-fold higher in DSB than DS (Figure 3J).

### 3.3. Changes in Reactive Oxygen Species (ROS) and Malondialdehyde (MDA)

**Figure 4:**
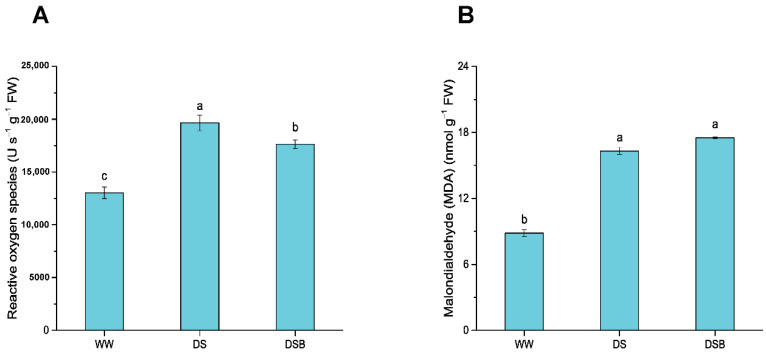
Oxidative stress response by tobacco plants to 2,4-epibrassinolide (EBR) application under drought stress. This figure comprises reactive oxygen species (**A**) and malondialdehyde content (**B**). The data are presented as means ± SE in the three treatments including WW (well-watered), DS (drought stress), and DSB (drought stress + 0.05 mM EBR application). Error bars represent SE of the mean; different letters on bars show significant differences at *p* < 0.05 using the LSD test.

The oxidative burst parameters were significantly (*p* < 0.05) affected by drought stress (DS) under exogenous EBR application. The results showed that ROS was 51% higher in DS and 35% higher in DSB compared with WW. However, ROS levels were 10% lower in DSB than in DS (Figure 4A). The MDA content increased by 88% and 92% in DS and DSB, respectively, compared with WW (Figure 4B).

### 3.4. Changes in Antioxidant Enzyme Activities and Their Related Gene Expression

**Figure 5:**
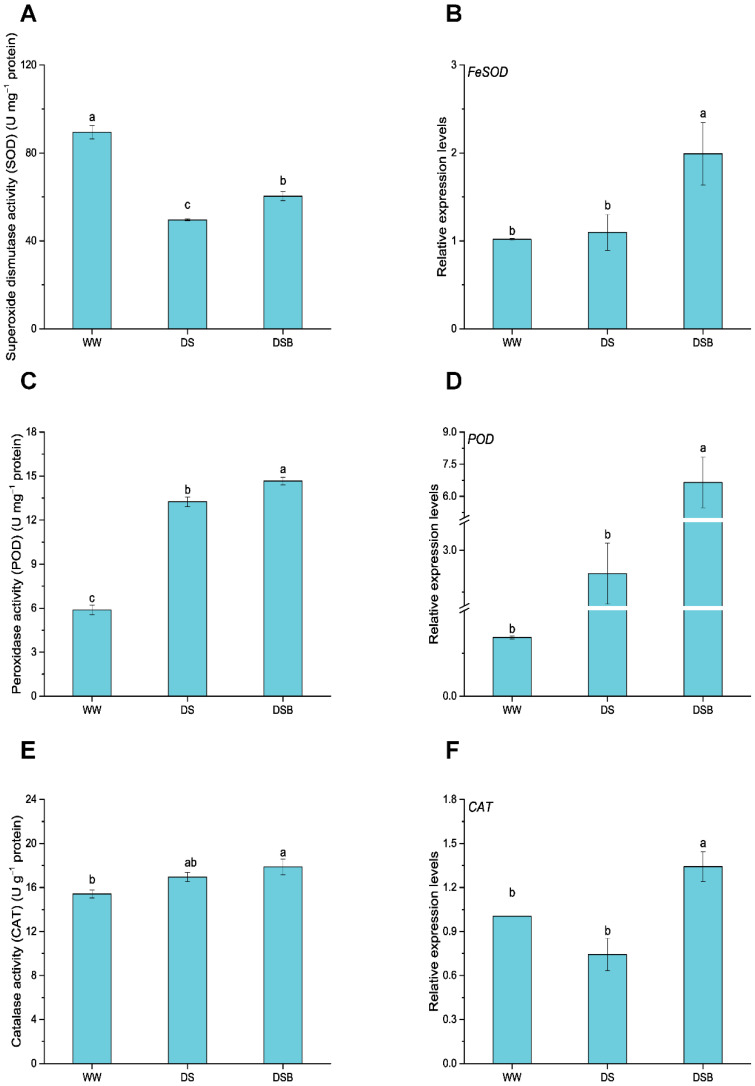
Antioxidant enzyme activities and their related gene expression as affected by 2,4-epibrassinolide (EBR) application under drought stress. This figure comprises SOD enzyme activity (**A**), *FeSOD* gene expression (**B**), POD enzyme activity (**C**), *POD* gene expression (**D**), CAT enzyme activity (**E**), and *CAT* gene expression (**F**). The data are presented as means ± SE in the three treatments including WW (well-watered), DS (drought stress), and DSB (drought stress + 0.05 mM EBR application). Error bars represent SE of the mean; different letters on bars show significant differences at *p* < 0.05 using the LSD test.

Antioxidant enzymes, including superoxide dismutase (SOD), peroxidase (POD), and catalase (CAT) activities and their related gene expression, were significantly (*p* < 0.05) affected by EBR application under DS (Figure 5). Of these enzyme activities, the SOD activity decreased by 45% and 32% in DS and DSB compared with WW, respectively; however, its activity was 22% higher in DSB compared with DS (Figure 5A). The gene related to the SOD enzyme (*FeSOD*) was upregulated in DSB by 2.0 and 1.8 times compared with WW and DS, respectively (Figure 5B). Furthermore, the POD activity increased in both DS (by 125%) and DSB (by 149%) treatments compared with WW; however, EBR application increased its activity by 11% compared with DS (Figure 5C). Similarly, the *POD* gene was also upregulated, and its transcript levels were 2.5 and 6.5 times higher in DS (nonsignificant) and DSB (significant) compared with WW, respectively, whereas its transcript levels were 2.6 times higher in DSB compared with DS (Figure 5D). Finally, CAT activity was enhanced by 10% in DS compared with WW, and was 16% and 5% higher in DSB compared with WW and DS, respectively (Figure 5E). The *CAT* gene was only upregulated in DSB, and its expression was 1.3 and 1.8 times higher compared with WW and DS (Figure 5F).

### 3.5. Changes in Osmoregulatory Substance Contents

**Figure 6:**
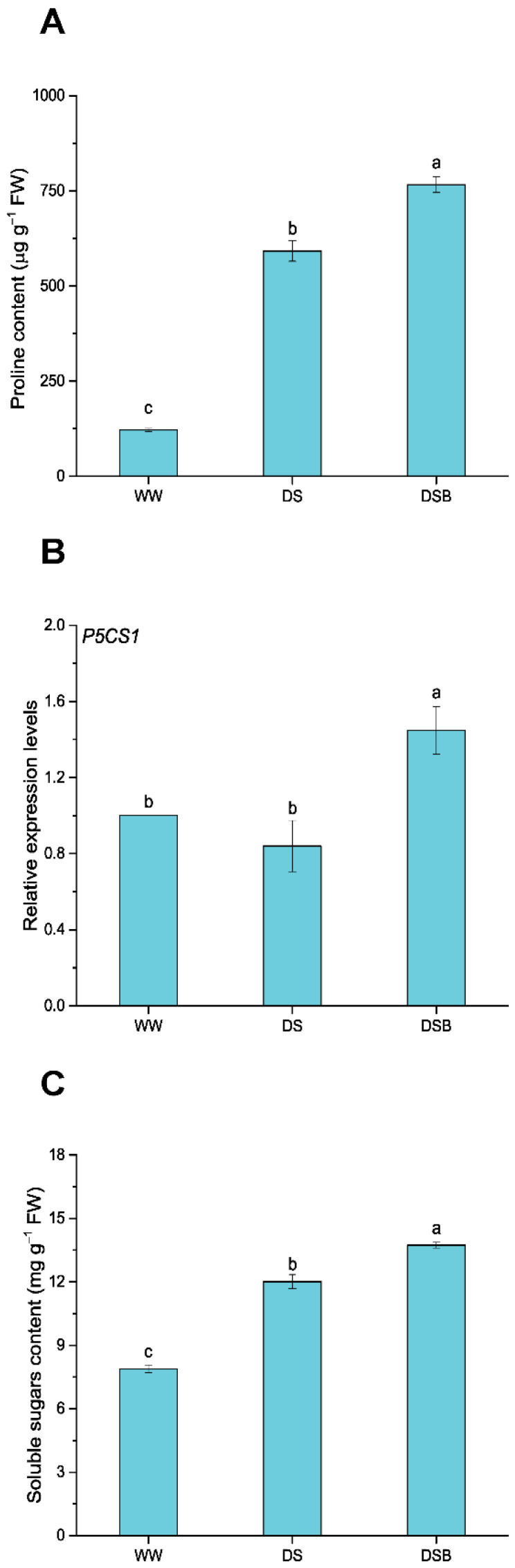
2,4-epibrassinolide (EBR) application improves the content of osmoregulatory substances under drought stress. This figure comprises proline content (**A**), *P5CS1*-proline biosynthesis gene expression (**B**), and soluble sugars content (**C**). The data are presented as means ± SE in the three treatments including WW (well-watered), DS (drought stress), and DSB (drought stress + 0.05 mM EBR application). Error bars represent SE of the mean; different letters on bars show significant differences at *p* < 0.05 using the LSD test.

The osmoregulatory substance (proline and soluble sugars (SS)) contents were also significantly (*p* < 0.05) affected by EBR application under DS. The proline content was augmented in both DS and DSB treatments compared with WW (Figure 6A). Results showed that the proline content increased from 121.58 μg g^−1^ FW in WW to 592.26 μg g^−1^ FW in DS and 766.14 μg g^−1^ FW in DSB. As a result, a 29% increase in proline content was observed in DSB compared with DS (Figure 6A). Proline biosynthesis genes, such as *P5CS1*, were also significantly upregulated in DSB, and its transcript levels were 1.4 and 1.7 times higher compared with WW and DS, respectively (Figure 6B). The SS was also augmented in both DS and DSB treatments compared with WW. SS levels increased from 7.88 mg g^−1^ FW in WW to 12.02 mg g^−1^ FW and 13.73 mg g^−1^ FW in DS and DSB, respectively. Overall, 14% more SS accumulated in DSB than DS (Figure 6C).

### 3.6. Changes in Phytohormones Content

**Figure 7:**
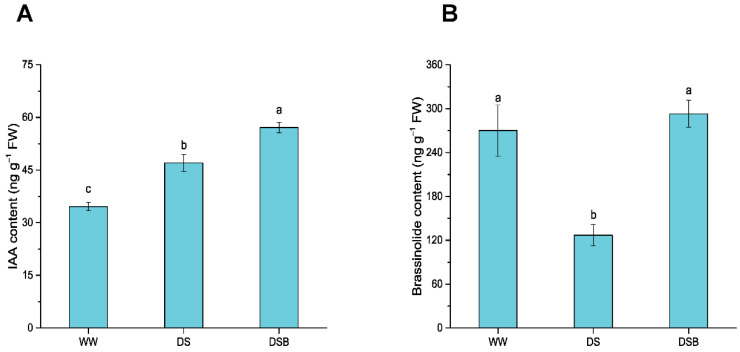
2,4-epibrassinolide (EBR) application improved phytohormone content, such as auxin (IAA) (**A**) and brassinolide content, in response to drought stress (**B**). The data are presented as means ± SE in the three treatments including WW (well-watered), DS (drought stress), and DSB (drought stress + 0.05 mM EBR application). Error bars represent SE of the mean; different letters on bars show significant differences at *p* < 0.05 using the LSD test.

EBR application significantly (*p* < 0.05) affected IAA and brassinolide (BL) contents under DS. The IAA content was augmented by EBR application and showed respective increases of 36% and 65% in DS and DSB compared with WW. Likewise, a 21% increase was also noted in DSB compared with DS (Figure 7A). The BL content significantly decreased under DS, whereas EBR application increased its content. The BL content decreased by 53% in DS compared with WW. However, its content increased by 131% in DSB compared with DS (Figure 7B).

### 3.7. Drought Tolerance-Related Gene Expression via BR and IAA Signaling Pathways

**Figure 8:**
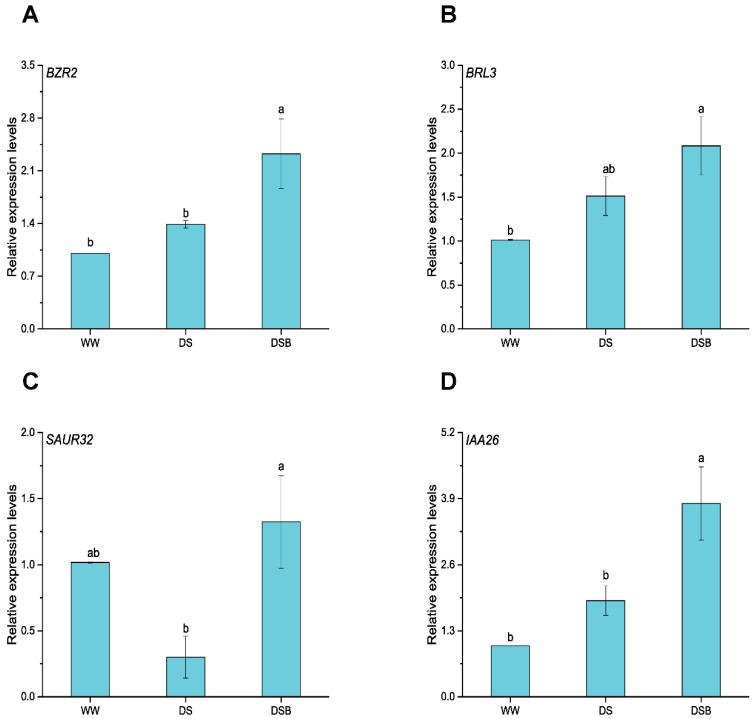
Drought tolerance-related gene expression is mediated by the BR and IAA signaling pathways. This figure displays gene expressions such as *BZR2* (**A**), *BRL3* (**B**), *SAUR32* (**C**), and *IAA26* (**D**). The data are presented as means ± SE in the three treatments including WW (well-watered), DS (drought stress), and DSB (drought stress + 0.05 mM 2,4-epibrassinolide application). Error bars represent SE of the mean; different letters on bars show significant differences at *p* < 0.05 using the LSD test.

Genes related to drought tolerance regulated by the BR (*BZR2* and *BRL3*) and IAA signaling pathways (*SAUR32* and *IAA26*) were significantly (*p* < 0.05) affected by EBR application under DS (Figure 8). *BZR2* was upregulated by 2.3 and 1.7 times in DSB compared with WW and DS, respectively (Figure 8A). Moreover, *BRL3* was upregulated by 2.1 and 1.4 times in DSB compared with WW and DS, respectively (Figure 8B). Finally, *SAUR32* and *IAA26* were significantly upregulated in DSB and showed respective 4.4- and 2.0- fold increases in expression compared with DS (Figure 8C,D).

## 4. Discussion

### 4.1. Tobacco Plants Adapted to Drought Stress by Promoting Leaf Thickness under 2,4-Epibrassinolide (EBR) Application

Thicker leaves thrive best under water-limited conditions, and leaf thickness is considered an important trait for plant functioning [10]. Anticlinal leaf growth is governed by individual cell layer growth, and tissue-specific growth is an important aspect of leaf growth regulation. Several studies indicate that drought stress (DS) reduces leaf thickness and the growth of individual cell layers. Drought stress reduced the total leaf thickness, with more reduction in mesophyll tissue thickness (thinner spongy and palisade cells) than in epidermal cells [47]. Similarly, another study found that drought stress reduced the total leaf thickness through decrease in cell size in individual cell layers, such as the upper and lower epidermis, palisade, and spongy layers [48]. In this study, leaf thickness and the thickness of palisade tissue, spongy tissue, and upper epidermis were decreased by drought stress, whereas EBR application under DS showed all tissue layers and ultimately increased leaf thickness. Under salt stress conditions, negative changes were observed in palisade and spongy tissues; however, EBR application increased the palisade and spongy tissue thickness and thus, the overall leaf thickness [11]. Cell elongation is controlled by various hormonal and environmental factors; *ARF6* (auxin-related hormonal transcription factor (TF)), *PIF4* (light and temperature regulated TF), and *BZR1* (brassinosteroid-signaling TF) interact with each other as a central-growth regulation circuit and control cell elongation [49]. Brassinosteroids are involved in cell elongation [50], and the treatment of plants/cells with brassinosteroid hormones controls the regulation of multiple plant developmental processes, including cell expansion [26]. Several studies have shown that brassinosteroid hormone application stimulated cell expansion and the thickness of various tissue-cell layers (palisade and spongy tissues) in plant species such as *Arabidopsis thaliana* [51], *Tabebuia alba* [52], and *Ficus carica* [53]. These findings support that EBR application increased leaf thickness, including the thickness of epidermal and mesophyll tissues under drought stress. Leaf thickness has been recognized as an indicator of drought tolerance [14], and drought-tolerant plants maintain higher leaf thickness [54]. Leaf thickness is a key trait that plants adapt for their survival and to survive under stressful conditions [55]. In this study, EBR improved leaf thickness and helped plants adapt to drought stress.

### 4.2. EBR Application Conferred Drought Tolerance via Higher Antioxidant Enzyme Activities and Osmoregulatory Substance Contents

**Figure 9:**
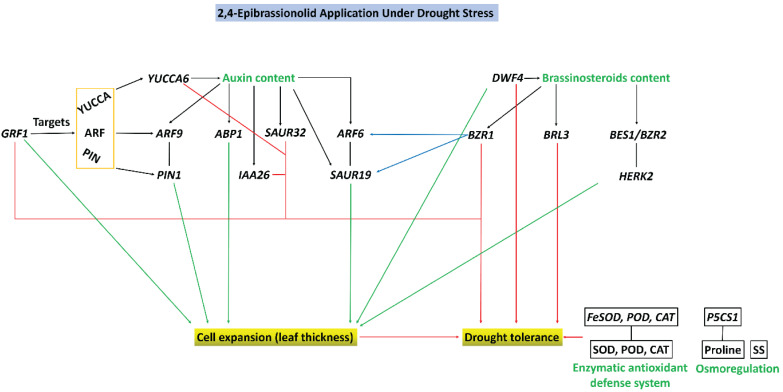
This schematic model shows the proposed mechanisms involved in drought tolerance regulation by 2,4-epibrassinolide (EBR) application. Integrative analyses found that EBR application regulated tolerance at anatomical, biochemical, and molecular levels under drought stress. Leaf thickness is increased by EBR under DS and improved drought tolerance and adaptation in tobacco plants. EBR application also conferred drought tolerance by improving the enzymatic antioxidant defense system and osmoregulation. BR and IAA crosstalk play crucial roles in drought tolerance. Black solid arrows show regulation. Green solid arrows indicate the regulation of cell expansion and, eventually, leaf thickness by the IAA and BR signaling pathways. Red solid arrows show the regulation of drought tolerance via the IAA and BR signaling pathways. Blue solid arrows show crosstalk between the IAA and BR signaling pathways.

The production of reactive oxygen species (ROS) in various cellular compartments is a fundamental process in plants, which keeps their levels stable by balancing them in the antioxidant defense system and setting the cell redox status [56]. Abiotic stresses, including drought stress, disturb this equilibrium and cause imbalances between ROS and the antioxidant defense system, leading to excessive ROS generation and inducing oxidative stress [8]. As a result of higher ROS production, the malondialdehyde (MDA) content increases (which shows the degree of plasma membrane damage) and is used as a biomarker for oxidative lipid injury [57,58]. To counter this increase in ROS content, plants activate antioxidant defenses that comprise antioxidant enzymes (superoxide dismutase—SOD, peroxidase—POD, catalase—CAT, ascorbate peroxidase—APX, glutathione reductase—GR, etc.), antioxidant substances (ascorbic acid—AsA, reduced glutathione—GSH), and osmoregulatory substance contents (proline and soluble sugars-SS) [59,60]. Cellular damage due to oxidative stress can also be prevented and minimized by accumulating compatible solutes (osmolytes); osmolytes safeguard cellular machinery via osmoregulation (accumulation of proline and SS) and protect cells from damage due to higher levels of ROS production [61]. On the one hand, reports revealed that drought stress increases ROS levels by engulfing antioxidant enzyme activities, such as decreased activities of SOD and CAT in rice [62], CAT in brassica [63], and CAT and POD in maize [64]. On the other hand, drought stress also enhances the enzymatic antioxidant defense system to reduce oxidative stress, such as higher activities of SOD, POD, and CAT in wheat [65], SOD and CAT in tomato plants [66], and SOD, POD, and CAT in oats [67]. In this study, drought stress decreased SOD activity, whereas CAT activity was statistically similar to WW. EBR application considerably alleviates the oxidative burst by decreasing ROS levels and increasing the activity of SOD, POD, and CAT under drought stress. Furthermore, our results found that EBR application improved antioxidant enzyme activities and scavenged ROS under drought stress [68,69,70]. Aside from higher antioxidant enzyme activities, their transcript levels (*FeSOD*, *POD*, and *CAT*) increased by EBR application under drought stress and play a role in conferring drought tolerance. The higher expression of *MnSOD*, *POD1*, and *POD3* in flax [71], *Cu/Zn-SOD* in common beans [72], *Cu/ZnSOD* and *CAT* in rice [73] under drought stress due to EBR application can also curb oxidative stress at gene transcript levels. The results of this study showed that EBR application under drought stress increased the activities of SOD, POD, and CAT and upregulated *FeSOD*, *POD*, and *CAT* genes, along with a higher accumulation of proline (*P5CS1* gene transcript levels) and SS (Figure 9), all of which decrease ROS levels and help tobacco plants to adapt to drought stress.

### 4.3. EBR Co-Ordinated Leaf Thickness and Conferred Drought Tolerance via Auxin and Brassinosteroids Signaling Pathways

Brassinosteroids (BR) and auxin (IAA) are two different classes of phytohormones that regulate plant growth and development by modulating and orchestrating cell division, cell elongation, and differentiation and help mitigate the adverse effects of abiotic stresses [74,75,76]. Apart from the individual involvement of BR and IAA in plant growth and development and response to abiotic stresses, hormonal crosstalk also modulates growth processes and response to abiotic stress [77], as the synergy between BR and IAA promotes cell elongation [78]. BR and IAA are involved synergistically in processes related to plant growth development, such as cell expansion, hypocotyl elongation, and vascular bundle development [18]. The *Arabidopsis* BR deficient mutant (*det2-1*) showed defects in hypocotyl elongation, similar to the response of auxin mutants to temperature stress, which highlights a possible functional interaction between BR and IAA for temperature-induced hypocotyl elongation [79]. Some studies established a connection (crosstalk) between BR and IAA related to their involvement in plant growth and developmental processes; however, further investigations have found that crosstalk is related to abiotic stress tolerance [80,81]. Therefore, in this study, the authors concluded that BR and IAA crosstalk could help improve drought tolerance.

Plants respond to drought stress by accumulating different phytohormones, including BR and IAA. These hormones further trigger drought tolerance by regulating several biochemical and molecular processes [82]. Several attempts have been made to highlight the role of genes involved in cell expansion/cell elongation induced by IAA. For example, *SAUR19* [83] and *ABP1* [84] induced by IAA signaling and *GRF1* [85] have been functionally validated and are involved in cell expansion. Similarly, the expression of *ARF9* and *PIN1* is supposedly involved in cell elongation in Chinese cabbage stalk [86]. Auxin efflux carriers (PIN family) mediate polar auxin transport that establishes a related auxin gradient and mediates developmental processes [87], such as cell division and cell expansion [88]. In this study, the higher expression of *SAUR19*, *ABP1*, *ARF9*, *PIN1*, and *GRF1* by exogenous application of EBR under drought stress (Figure 3) indicated the occurrence of cell expansion, which resulted in higher leaf thickness. IAA is involved in cell expansion, and recent studies have demonstrated that they are also involved in abiotic stress responses [89]. *SAUR* and *AUX/IAA* are some of the key auxin-responsive genes [90]. *IAA6* in Rice controls tiller growth and confers drought tolerance [91], and *SAUR32* is also involved in drought stress adaptation [92]. Similarly, overexpression of the IAA biosynthesis gene (*YUCCA6*) also enhanced drought tolerance [93,94]. The *GRF1* also targets the auxin biosynthesis, transport, and signaling genes [95] and plays a crucial role in maintaining plant fitness under stressful conditions. The exogenous application of EBR upregulated the expression of *YUCCA6*, *IAA6,* and *SAUR32* under drought stress, which provides evidence for their involvement in conferring drought tolerance in tobacco (Figure 3, Figure 8, and Figure 9).

Apart from the role of IAA in cell expansion and drought tolerance, the role of BR at both exogenous and endogenous levels must be recognized. Previous studies confirmed that BR is involved in cell expansion [96], positively regulates abiotic stress responses, and improves drought tolerance [97,98]. *DWF4*, a BR biosynthesis gene, is upregulated, and ultimately higher BL contents were observed in drought-stressed plants treated with EBR. *DWF4* (*CYP90*) encodes cytochrome P450 involved in BR biosynthesis [99,100] and is also involved in cell elongation [101]. The *HERK2* gene is involved in cell elongation and is regulated by *BES1* (*BZR2*) [102]. Similarly, *BES1* is accumulated in the nucleus and regulates gene expression, and promotes stem elongation in response to BR [103]. It has been speculated that EBR application promotes leaf thickness by inducing cell elongation in mesophyll cells (palisade and spongy tissues) under drought stress, which could be due to upregulation of the *DWF4*, *BZR2*, and *HERK2* genes (Figure 3). Furthermore, endogenous BR content is correlated with various degrees of drought sensitivity, and drought-tolerant genotypes accumulate higher BR contents [104], so in this study, we observed higher BL amounts (Figure 7). *BRL3* (brassinosteroid receptor) overexpression confers drought tolerance without affecting plant growth via higher proline and soluble sugars accumulation [105]. Similarly, *BZR2* in wheat also improved drought tolerance via ROS scavenging antioxidant enzyme-related gene expression [106]. In another study, the authors proposed that higher expression of *DWF4* and *BZR2* genes show their roles in response to drought stress [107]. The expression of *DWF4*, *BRL3*, and *BZR2* is higher in drought-stressed plants treated with EBR, indicating their roles in conferring drought tolerance and could also strengthen the enzymatic antioxidant plant defense system and improve osmoregulation.

Both phytohormone signals can encourage cell expansion, and BR and IAA are two master regulators that have additive effects on plant growth and development [18]. These two hormones also control a number of other growth processes, including cell elongation. This suggests crosstalk between the different pathways [108]. In *Arabidopsis thaliana*, ARF and BZR are interdependent in promoting hypocotyl elongation, as *ARF6/8* are required for *BZR1* promotion of hypocotyl elongation and regulate a core set of genes involved in cell elongation [49]. Similarly, in another study, BR promotes the expression of *SAUR19* via *BZR1,* and as mentioned earlier, *ARF6* and *BZR1* interact with each other [49] and synergistically induce the expression of SAURs (especially S*AUR19*), which promotes cell expansion [109]. In this study, *BZR1*, *ARF6*, and *SAUR19* genes are expressed by EBR application under drought stress, inducing cell expansion and promoting leaf thickness. This demonstrates crosstalk between BR and IAA (Figure 9).

## 5. Conclusions

In conclusion, drought stress negatively affected anatomical and biochemical characteristics, along with gene expression in tobacco plants. However, the application of 2,4-epibrassinolide (EBR) can significantly ameliorate the effects of drought stress. Drought stress decreased leaf thickness, whereas EBR application increased leaf thickness. EBR application promoted leaf thickness via higher transcript levels of genes related to cell expansion induced by the IAA (*ARF9*, *ARF6*, *PIN1*, *SAUR19*, and *ABP1*) and BR (*DWF4*, *HERK2*, and *BZR1*) signaling pathways, contributing to drought tolerance. Additionally, EBR application conferred drought tolerance via an improved enzymatic antioxidant defense system and osmoregulation. EBR application also upregulated genes related to drought tolerance that are also induced by the IAA (*YUCCA6*, *SAUR32*, and *IAA26*) and BR (*BRL3* and *BZR2*) signaling pathways. Therefore, crosstalk between the BR and IAA signaling pathways is important in drought tolerance in tobacco plants.

## Data Availability

The data presented in this study are available on request from the corresponding author.

## Supplementary Materials

The following supporting information can be downloaded at: https://www.mdpi.com/article/10.3390/biology11081192/s1, Table S1. Primers list for RT-qPCR.
