# Supplementary material for: Application of 2,4-Epibrassinolide Improves Drought Tolerance in Tobacco through Physiological and Biochemical Mechanisms
Source: Biology (Basel). 2022 Aug 8;11(8):1192. doi: 10.3390/biology11081192 (PMC9405153; doi:10.3390/biology11081192)
Supplement: Supplementary file 1 [file biology-11-01192-s001.zip › biology-1814078-supplementary.pdf]

Application of 2,4-Epibrassinolide Improves Drought Tolerance in Tobacco Through Physiological and Biochemical Mechanisms

Rayyan Khan, Xinghua Ma*, Quaid Hussain, Muhammad Asim, Anas Iqbal, Xiaochun Ren, Shahen Shah, Keling Chen, Yi Shi

* **Correspondence:** maxinghua@caas.cn

Table S1: Primers list for RT-qPCR

Gene Name	Forward Primer (5′-3′)	Reverse Primer (5′-3′)
YUCCA6	TAGCCGATGGTTGATCGTGG	TCCAAGCACACCTCCATTCC
ARF9	TAGAGGCCAACCTCGGAGAC	GAGCAAGCCGTCTAACACCA
PIN1	CTGTTGGCCTTCGTGGAGTT	CCGTGCTAAGAATGTCAGGGT
ABP1	CTCGCCATGTTCTCGTAGTGG	CCTGTGGAAGCTCGCTGAT
SAUR19	GAGAGAGCCAGAAGAAGCGA	CAAGCGGGAGGTGAGATCAAT
GRF1	TAGCCACGGTGAACCTATGC	GGGCTGCTATCCCACATCTC
SAUR32	GTGTCTGGCAATTACTGTGGG	AGCTTCCTTGTCAATCACCCC
IAA26	AAGCTGGAGTTAAGGCTTGGT	TGCTGATATTGTAACCAAAAGGGA
ARF6	CCCAATAGGTGGAATGCAAATG	CGAAGTGAGAGCCTGTTTAGAG
DWF4	AAATGATGGTGGAAGCTGCTGT	GTCTCGTAACCACCTAGTAAGGAA
HERK2	CGATGCGGTTTCAACGACAA	GTTGGTCTGCTCTTCCCCTC
BZR2	ATTGGTGGTTCGGCATCAGT	GAGACTGGGCTGGGGATAGA
BZR1	CATCTTATCAGCCGAGTCTAACC	TGGAGGAAGTGATGAAGGAATG
BRL3	CGAAGGGGAGAAAGGAGGGAT	TTACCAGCCTTGCCATTCCA
FeSOD	ACCCTCAAGGCTATCAAATGTTAAG	ACGATGATGCTCTCCCCAAT
POD	GCTCATTCTTCTTCTTTTGCCGTT	GAGGCTAGCCCCAATACGAG
CAT	CAATGTGCACTGGCAAACGA	GATCAGACAAGGCCTCCACC
P5CS1	AGTGGCCCTCCCCGTAATCC	GAAGAGGGTGCCGATACATTCC
Actin	CAAGGAAATCACCGCTTTGG	AAGGGATGCGAGGATGGA
